# The Number of Traditional Cardiovascular Risk Factors Is Independently Correlated with Disease Activity in Patients with Psoriatic Arthritis

**DOI:** 10.3390/medicina56080415

**Published:** 2020-08-18

**Authors:** Iván Ferraz-Amaro, Diana Prieto-Peña, Natalia Palmou-Fontana, David Martínez-López, Laura de Armas-Rillo, Alicia García-Dorta, Belén Atienza-Mateo, Ricardo Blanco, Susana Armesto, Miguel Á. González-Gay

**Affiliations:** 1 Division of Rheumatology, Hospital Universitario de Canarias, 38320 Tenerife, Spain; iferrazamaro@hotmail.com (I.F.-A.); alicia.garcia.dorta@gmail.com (A.G.-D.); 2 Epidemiology, Genetics and Atherosclerosis Research Group on Systemic Inflammatory Diseases, Division of Rheumatology, Hospital Universitario Marqués de Valdecilla, IDIVAL, 39011 Santander, Spain; diana.prieto.pena@gmail.com (D.P.-P.); npalmou@gmail.com (N.P.-F.); david200999@hotmail.com (D.M.-L.); mateoatienzabelen@gmail.com (B.A.-M.); rblanco@humv.es (R.B.); 3 Division of Health Sciences. Universidad Europea de Canarias, 38300 Tenerife, Spain; laura.spm@gmail.com; 4 Dermatology, Hospital Universitario Marqués de Valdecilla, IDIVAL, 39011 Santander, Spain; susana.armesto@scsalud.es; 5 School of Medicine, University of Cantabria, 39011 Santander, Spain; 6 Cardiovascular Pathophysiology and Genomics Research Unit, Faculty of Health Sciences, School of Physiology, University of the Witwatersrand, Johannesburg 2000-2199, South Africa

**Keywords:** psoriatic arthritis, cardiovascular risk factors, disease activity, cardiovascular disease, atherosclerosis

## Abstract

*Background and objectives:* Psoriatic arthritis (PsA) is associated with several comorbidities, including among others an increased risk of cardiovascular (CV) disease, atherosclerosis, metabolic syndrome, hypertension dyslipidemia, and diabetes. The purpose of the present study was to determine how the number of CV risk factors correlates with disease related data such as disease activity. *Materials and Methods:* Cross-sectional study that encompassed 305 patients who fulfilled the CASPAR criteria for PsA were assessed for lipid profile, disease activity measurements, and the presence of six traditional CV risk factors (diabetes mellitus, dyslipidemia, hypertension, obesity, chronic kidney disease, and smoking status). A multivariable regression analysis, adjusted for age, sex, and disease duration, was performed to evaluate if the number of classic CV risk factors was independently related with specific features of the disease, including disease activity. *Results:* Disease duration was found to be higher, after adjustment for age and sex, in patients with 1 or 2, and 3 or higher CV factors, compared to those patients without CV risk factors. Similarly, DAPSA (Disease Activity in PSoriatic Arthritis score) was found to be independently upregulated in patients with a higher number of CV risk factors. In this sense, as DAPSA score increases the odds ratio (OR) of having 1 or 2 (OR 1.12 (95% confidence interval (CI) 1.03–1.21), *p* = 0.010), and 3 or higher (OR 1.15 (95% CI 1.04–1.26), *p* = 0.004) CV factors was significantly higher compared to no CV risk factors category. This was independently found after adjustment for age, sex, and disease duration. *Conclusions*: PsA patients with a higher number of CV risk factors exhibit an upregulated disease activity compared to those without them. This is independent of disease duration and other demographics factors.

## 1. Introduction

The five leading modifiable risk factors (hypercholesterolemia, diabetes, hypertension, obesity, and smoking) are estimated to be responsible for more than half of cardiovascular (CV) mortality in the general population [1]. Accordingly, the absence of these major risk factors predicts a much lower risk of coronary heart disease [2]. It is also accepted that after adjusting for age and sex, the odds of CV disease increases with the number of risk factors present (OR 2.4, 4.2, 4.9, and 7.2 for 1, 2, 3, and 4 or more risk factors, respectively, compared with no risk factors) [3].

Psoriatic arthritis (PsA) is associated with an increased risk of CV disease [4,5] and CV events [6]. Besides, PsA often exhibits an increased risk of metabolically linked comorbidities such as obesity, insulin resistance, type 2 diabetes mellitus, non-alcoholic fatty liver disease, dyslipidemia, and others [7]. For example, metabolic syndrome [8] and diabetes [9] are more common among patients with PsA than the general population and are also associated with disease severity. Although most of these studies have the limitation that they are cross-sectional, these increased comorbidities have been attributed to the inflammatory burden that patients with PsA have [10].

Low-grade chronic inflammation is now widely accepted to be associated with CV risk factors in general population. For example, diabetes mellitus and adiposity have been correlated with increased levels of markers of inflammation, including C-reactive protein, interleukin-6, plasminogen activator inhibitor-1, tumor necrosis factor-alpha, and white cell count [11]. A link between CV disease, inflammation, and traditional CV risk factors exist. In this regard, chronic inflammation favors the development of metabolic abnormalities included within the traditional CV risk factors. On the other hand, these CV risk factors are also associated with the presence of underlying chronic inflammation.

Previous studies regarding CV disease in PsA have been mainly focused on how inflammation affects subclinical CV disease or CV events. However, the way in which CV risk factors correlate with disease related data in PsA has not been extensively explored. If this were the case, not only the inflammation would cause CV disease, but, in turn, the associated comorbidity would modify per se the inflammatory state in patients with PsA. Taking all this into account, we aimed to assess in this cross-sectional study whether patients with a higher number of CV risk factors may have a higher disease activity or if they express differential features related to the disease.

## 2. Methods

### 2.1. Study Participants

This was a cross-sectional study that included 305 patients with PsA and 179 controls. All of them were 18 years old or older, had a clinical diagnosis of PsA, and were enrolled based upon the international Classification of Psoriatic Arthritis (CASPAR) study [12]. They had been diagnosed by rheumatologists and were periodically followed-up at rheumatology outpatient clinics. For the purpose of inclusion in the present study, PsA disease duration had to be ≥1 year. Those undergoing anti-TNF-α, IL-17 inhibitors or other biological therapies were not excluded from the present study. Likewise, since glucocorticoids are often used in the management of PsA, patients taking prednisone were not excluded. None of the patients had established CV disease. The study protocol was approved by the Institutional Review Committee at Hospital Marqués de Valdecilla in Santander, Spain, and all subjects provided informed written consent (Approval Number: 2016.052).

### 2.2. Assessments and Data Collection

Surveys in PsA patients were performed to assess CV risk factors and medication. Hypertension was defined as a systolic or a diastolic blood pressure higher than 140 and 90 mmHg, respectively. Dyslipidemia was defined if one of the following factors was present: total cholesterol >200 mg/dL, triglycerides >150 mg/dL, HDL-cholesterol <40 mg/dL in men or <50 mg/dL in women, or LDL-cholesterol >130 mg/dL. Chronic kidney disease was defined as a glomerular filtration rate <60 mL/min/1.73 m^2^. At the time of assessment, all patients were evaluated using two clinical measures of disease activity: Bath Ankylosing Spondylitis Disease Activity Index (BASDAI) [13] and the Disease Activity Index for Psoriatic Arthritis (DAPSA) [14]. Additionally, patients with PsA were defined as being in clinical remission (DAPSA < 4) or having low (DAPSA in the range of 5 to 14), moderate (DAPSA of >15 to 28), or high disease activity (DAPSA > 28) as previously described [14]. In addition, a functional status index (Bath Ankylosing Spondylitis Functional Index (BASFI)) [15], a patient life impact measure (PsA Impact of Disease Score (PsAID)) [16], two cutaneous indexes (Psoriasis Area and Severity Index score (PASI) and Psoriasis Global Assessment (PGA)) [17], and the Nail Psoriasis Severity Index (NAPSI) were used to assess the severity of nail psoriasis [18]. Furthermore, high-sensitivity C-reactive protein (hsCRP) was assessed, and standard techniques were used to measure serum lipids.

### 2.3. Statistical Analysis

Demographic and clinical characteristics in patients with PsA were described as mean ± standard deviation or percentages for categorical variables. For non-normally distributed continuous variables, data were expressed as a median and interquartile range (IQR). Univariate differences between patients with 0, 1, or 2, and 3 or more CV risk factors were assessed through ANOVA or Kruskall–Wallis tests according to normal distribution or the number of subjects. Multinomial logistic regression analysis adjusted for age, sex, and disease duration was performed to assess the relation of disease characteristics and number of CV risk factors. Patients with zero CV risk factors were considered the reference category, being patients with 1 or 2, and 3 or more CV risk factors, compared to this reference category. All the analyses used a 5% two-sided significance level and were performed using SPSS software, version 24 (IBM, Chicago, IL, USA). A *p* value < 0.05 was considered statistically significant.

## 3. Results

### 3.1. Demographic and Disease-Related Data

A total of 305 PsA patients with a mean ± SD age of 54 ± 12 were included in this study. The frequency and percentages of the six CV risk factors, and demographic and disease-related characteristics of the participants, are shown in Table 1. While 27% (*n* = 82) of the patients did not have CV risk factors, the presence of 1–2, and 3 or more CV risk factors was observed in 52% (*n* = 160) and 21% (*n* = 63) of patients, respectively.

The median PsA disease duration was 6 years (IQR 3–13). Psoriasis was present in 70% of patients at the time of the study and 10% were positive for HLA-B27. Most PsA patients were in the remission activity category, as shown by the DAPSA score (median 3.8 (IQR 0.0–12.6)). BASDAI total score was 2.2 (IQR 0.0–4.6) and 10% of the patients had a BASDAI higher or equal to 4. About half of the patients (45%) were taking prednisone and 79% were on NSAIDs. Seventy three percent of patients were using DMARDs, methotrexate was reported in 68% of the subjects, and 42% were receiving TNF-alpha inhibitors at the time the study was performed. Additional PsA information is shown in Table 1.

### 3.2. Number of CV Risk Factors and Association with Disease Related Data and Disease Activity Scores

Univariable differences regarding demographics and PsA related data between patients with 0, 1–2, and 3 or more CV risk factors, is shown in Table 2. Age and the presence of male gender were different between groups. In this regard, those with more CV risk factors were older. Similarly, disease duration and the presence of axial and peripheral symptoms differed between groups. Interestingly, DAPSA score was higher in individuals with more CV risk factors (Table 2).

When these differences were assessed through multivariable multinomial regression analysis, using non-CV risk factors as the reference variable, some differences were observed. (Table 3). Disease duration was found to be positively and independently associated with a higher odds ratio—OR—of having 1–2 CV risk factors (OR 1.06 (95% CI 1.04–1.08), *p* = 0.000), and ≥3 CV risk factors (OR 1.12 (95% CI 1.09–1.16), *p* = 0.000) compared to those patients without CV risk factors (after adjustment for age and sex). Similarly, DAPSA score was found to be positively associated with a higher number of CV risk factors. In this sense, as DAPSA score increases, the OR of having a higher number of CV risk factors was significantly greater (OR 1.12 (95% CI 1.03–1.21), *p* = 0.010 for 1–2 CV risk factors; and OR 1.15 (95% CI 1.04–1.26), *p* = 0.004 for ≥3 CV risk factors), compared to the no CV risk factors category. This was independently found after adjustment for age, sex, and disease duration. When these differences were assessed using DAPSA score categories, patients within the low and moderate or high DAPSA score category showed significant odds for the presence of 1–2 CV risk factors compared to those who were in remission category. Similar results were found for the odds of 3 or more CV risk factors, although, in this case, results did not reach statistical significance. No other relations were found between disease-related data and the number of CV risk factors (Table 3).

## 4. Discussion

Cardiometabolic comorbidities represent a considerable burden in patients with PsA. Although our study was cross-sectional and, therefore, causality cannot be inferred, patients with higher number of CV risk factors show greater disease activity assessed by DAPSA score. According to these findings, the presence of several CV risk factors may be correlated with an increased systemic inflammatory burden that, in turn, may favor a superior disease activity.

CV risk factors are highly overrepresented in patients with PsA compared to matched subjects from the general population [19]. In our series, 73% of patients showed 1 or more CV risk factors. Apart from CV comorbidities, PsA is also associated with other comorbid conditions such as anxiety, fatigue, smoking habit, alcohol consumption, overweight and obesity, depression, and osteoporosis [20]. These associated comorbidities may enhance the CV risk of these patients.

Information regarding the additive effect of traditional CV risk factors on disease activity in patients with PsA is scarce. Most studies about comorbidity in PsA focused on non-CV features. In a recent report on the association of comorbidity with quality of life, the Rheumatic Disease Comorbidity Index (an index that includes lung disease, CV disease, fracture, depression, diabetes, cancer, and stomach ulcer problem), was significantly related to anxiety [21]. In this study, the type of comorbidity appeared to have a greater impact than the number of comorbidities. In the cohort of Danish patients with PsA (DANBIO), the presence of comorbidities, assessed by the Charlson Comorbidity Index, was associated with higher baseline disease activity, shorter anti tumor necrosis factor therapies persistence, and reduced clinical response rates [22]. Moreover, obesity has been associated with a lower probability of achieving sustained minimal disease activity among patients with PsA [23]. In another study of patients with either psoriasis or PsA, almost 88% of patients had at least one modifiable CV risk factor: 17% were current smokers, 13% had type 2 diabetes mellitus, 45% had hypertension, almost 50% had dyslipidaemia, and >75% were overweight or obese [24]. However, this study did not assess the relation of this CV comorbidity with PsA related features. Interestingly, a recent study of our group showed disease activity measured by DAPSA influenced the CV risk reclassification based on carotid ultrasound in patients with PsA [4].

One limitation of our study was that we could not establish the actual impact on each individual CV risk factor on disease activity. In this regard, we could not determine the interaction between them of several CV risk factors. We additionally acknowledge that we did not record the duration or severity of the CV factors, or if they were present before or after the diagnosis of the disease. However, we believe, that due to our sample size, the entire spectrum of duration or severity of the CV risk factor has probably been captured. Moreover, quantity of previous DMARDs or anti TNF-alpha treatments, as well as cumulative glucocorticoid dosage, were not assessed in our study. For this reason, we cannot conclude whether the amount of previous treatments had any relation with the number of CV risk factors. Nevertheless, we believe this did not affect the main result of our study regarding the association between DAPSA and the number of CV risk factors.

In our study population, the use of steroids was high. We do not have an exact explanation for this. We believe this is due to certain characteristics of our patients, such as the severity of the disease, or perhaps, to our own clinical practice that includes the use of steroids in routine habits. In any case, no relationship between the number of CV risk factors and the use of steroids was found in our work. We acknowledge that the use of steroids could have made the number of CV risk factors higher or that they were more serious. Nevertheless, as prednisone use was not associated with the number of CV risk factors in the univariable analysis, no adjustment was needed in the multivariable relation of DAPSA with them.

However, our study has some strengths derived from the monocentric design, with the inclusion of a well-defined cohort of patients homogeneously evaluated and followed by clinicians highly experienced in PsA. In addition, to the best of our knowledge, our study constitutes the first attempt to study exclusively CV comorbidity and its relationship with disease features. According to our results, patients with more CV risk factors were more frequently male and older. The multivariable analysis revealed that the number of CV risk factors, as the independent variable, was independently associated with disease activity. Although, the interconnection of traditional CV risk factors and its role in developing CV disease is not completely understood, we believe that the additive effect of several CV risk factors may favor a higher inflammatory state that, in turn, may promote a higher disease activity.

In conclusion, in our study the number of CV risk factors was correlated with a poorer disease activity in patients with PsA. Increasing awareness of the influence of CV risk factors in PsA, not only on the CV disease, but also on disease activity appears to be of major relevance.

## Figures and Tables

**Table 1:** Data of the 305 psoriatic arthritis patients.

	Psoriatic Arthritis
	(*n* = 305)
Demographics	
Male, *n* (%)	141 (46)
Age, years	54 ± 12
BMI, mg/cm^2^	27 ± 7
Waist circumference, cm	94 ± 17
Systolic pressure, mmHg	138 ± 21
Diastolic pressure, mmHg	79 ± 12
Comorbidity	
Hypertension, *n* (%)	108 (35)
Dyslipidemia, *n* (%)	157 (51)
Current smoking, *n* (%)	76 (25)
Diabetes, *n* (%)	33 (11)
BMI > 30, *n* (%)	51 (17)
Chronic kidney disease, *n* (%)	9 (3)
Number of CV risk factors	
0	82 (27)
1–2	160 (52)
>= 3	63 (21)
SCORE	1.0 (0.3–3.1)
Analytical data	
ESR, mm/h	6 (3–13)
CRP, mg/L	0.2 (0.1–0.7)
Cholesterol, mg/dL	189 ± 38
Triglycerides, mg/dL	102 ± 50
LDL, mg/dL	111 ± 34
HDL, mg/dL	57 ± 17
Atherogenic index	3.9 ± 5.6
Psoriatic arthritis related data	
Type of psoriatic arthritis	
Peripheral oligoarthritis	29 (10)
Peripheral polyarthritis	162 (53)
Spondylitis	43 (14)
Mixed	54 (18)
Disease duration, years	5 (2–11)
Psoriasis, *n* (%)	213 (70)
HLA-B27, *n* (%)	29 (10)
Positive family history of PsA, *n* (%)	77 (25)
BASDAI, total score	2.2 (0.0–4.6)
BASDAI >4, *n* (%)	32 (10)
BASFI, total score	0 (0–3)
PsAID, total score	1.0 (0.0–2.8)
DAPSA, total score	3.8 (0.0–12.6)
BSA, total score	0.7 (0.0–2.1)
PASI, total score	0.4 (0.0–2.0)
NAPSI, total score	0 (0–3)
PGA, total score	0 (0–1)
Axial symptoms, *n* (%)	101 (33)
Peripheral symptoms, *n* (%)	213 (70)
Hip symptoms, *n* (%)	55 (18)
Enthesitis, *n* (%)	111 (36)
Uveitis, *n* (%)	19 (6)
Dactylitis, *n* (%)	79 (26)
Inflammatory bowel disease, *n* (%)	16 (5)
Sacroiliitis on MRI, *n* (%)	33 (11)
Syndesmophytes in axial X-ray, *n* (%)	12 (4)
Current NSAIDs, *n* (%)	240 (79)
Current prednisone, *n* (%)	137 (45)
DMARDs, *n* (%)	224 (73)
Methotrexate, *n* (%)	208 (68)
Anti TNF therapy, *n* (%)	129 (42)

Data represent means ± SD or median (IQR) when data were not normally distributed. BMI: body mass index; CRP: C reactive protein. DMARD: disease-modifying antirheumatic drug; ESR: erythrocyte sedimentation rate. HDL: high-density lipoprotein; LDL: low-density lipoprotein. BASFI: Bath Ankylosing Spondylitis Functional Index. BASDAI: Bath Ankylosing Spondylitis Disease Activity Index. NAPSI: Nail Psoriasis Severity Index; BSA: Body Surface Area. PGA: Psoriasis Global Assessment; PsAID: PsA Impact of Disease Score. DAPSA: Disease Activity in PSoriatic Arthritis. Chronic kidney disease is defined as a glomerular filtration rate <60 mL/min/1.73 m^2^. MRI: Magnetic Imaging Resonance.

**Table 2:** Number of CV risk factors and association with disease related data and disease activity scores.

	Number of CV Risk Factors			
	0	1–2	≥3	
	(*n* = 82)	(*n* = 160)	(*n* = 63)	
Demographics				*p*
Male, *n* (%)	44 (54)	63 (39)	34 (54)	**0.042**
Age, years	47 ± 10	54 ± 12	60 ± 9	**0.000**
ESR, mm/h	5 (2–12)	6 (3–13)	7 (4–15)	0.12
CRP, mg/dL	0.3 (0.1–0.7)	0.2 (0.1–0.5)	0.3 (0.1–0.8)	0.88
Psoriatic arthritis related data				
Disease duration, years	5 (3–10)	4 (1–10)	6 (2–17)	**0.035**
HLA-B27, *n* (%)	11 (13)	16 (10)	2 (3)	0.18
BASDAI, total score	1.4 (0.0–3.4)	2.7 (0.0–4.6)	2.2 (0.0–5.5)	0.57
BASDAI >4, *n* (%)	6 (7)	20 (13)	6 (10)	0.47
BASFI, total score	0 (0–2)	0 (0–3)	1 (0–3)	0.20
PsAID, total score	1.0 (0.0-2.3)	1.4 (0.0–3.2)	0.1 (0.02.3)	0.32
**DAPSA, total score**	**0.2 (0.0**–**6.5)**	**4.7 (0.0**–**15.6)**	**6.1 (0.1**–**15.0)**	**0.014**
Remission, *n* (%)	19 (23)	32 (20)	13 (21)	0.076
Low, *n* (%)	7 (9)	16 (10)	8 (13)	
Moderate or high, *n*(%)	1 (1)	18 (11)	8 (13)	
BSA, total score	0.0 (0.0–1.6))	0.7 (0.0–2.4)	1.3 (0.0–2.0)	0.44
PASI, total score	0.0 (0.0–1.8)	0.4 (0.0–2.5)	1.6 (0.2–2.4)	0.17
NAPSI, total score	0.0 (0.0–2.8)	0.0 (0.03.0)	0.9 (0.0–6.0)	0.64
PGA, total score	0.0 (0.0–1.0)	0.0 (0.0–1.0)	1.0 (0.02.0)	0.31
Axial symptoms, *n* (%)	32 (39)	56 (35)	13 (20)	**0.025**
Peripheral symptoms, *n* (%)	46 (56)	124 (78)	43 (68)	**0.017**
Hip symptoms, *n* (%)	15 (18)	27 (17)	13 (21)	0.72
Enthesitis, *n* (%)	29 (35)	63 (39)	19 (30)	0.38
Uveitis, *n* (%)	4 (5)	9 (6)	6 (10)	0.54
Dactylitis, *n* (%)	21 (26)	37 (23)	21 (33)	0.22
Inflammatory bowel disease, *n* (%)	3 (4)	10 (6)	3 (5)	0.78
Sacroiliitis on MRI, *n* (%)	11 (13)	12 (8)	9 (14)	0.13
Syndesmophytes in X-ray, *n* (%)	3 (4)	7 (4)	2 (3)	0.96
Current NSAIDs, *n* (%)	60 (73)	133 (83)	47 (75)	0.50
Current prednisone, *n* (%)	36 (44)	70 (44)	31 (49)	0.42
DMARDs, *n* (%)	53 (65)	120 (75)	51 (81)	0.45
Methotrexate, *n* (%)	47 (57)	116 (73)	45 (71)	0.35
Anti TNF therapy, *n* (%)	35 (43)	73 (46)	21 (33)	0.13

Data represent means ± SD or median (IQR) when data were not normally distributed. CRP: C reactive protein; ESR: erythrocyte sedimentation rate. DMARD: disease-modifying antirheumatic drug; NSAIDs: Nonsteroidal anti-inflammatory drugs. BASFI: Bath Ankylosing Spondylitis Functional Index. BASDAI: Bath Ankylosing Spondylitis Disease Activity Index. NAPSI: Nail Psoriasis Severity Index; BSA: Body Surface Area. PGA: Psoriasis Global Assessment; PsAID: PsA Impact of Disease Score. DAPSA: Disease Activity in PSoriatic Arthritis. DAPSA categories were defined as: remission (DAPSA <4), low (DAPSA in the range of 5 to 14), moderate (DAPSA of >15 to 28) or high disease activity (DAPSA >28). *p* values lower than 0.05 are depicted in bold.

**Table 3:** Multivariable analysis of the association of disease related data with number of CV risk factors.

	Number of CV Risk Factors		
	Odds Ratio (95% CI), *p*		
	0	1–2	>=3
	(*n* = 82)	(*n* = 160)	(*n* = 63)
Disease duration, years	-	**1.06 (1.04–1.08), 0.000**	**1.12 (1.09–1.16), 0.00**
ESR, mm/1st h	-	1.01 (0.96–1.07), 0.64	1.04 (0.98–1.11), 0.20
CRP, mg/L	-	1.01 (0.80–1.28), 0.90	0.97 (0.74–1.28), 0.97
HLA-B27, *n* (%)	-	0.98 (0.32–2.03), 0.97	1.68 (0.26–10.80), 0.58
BASDAI, total score	-	1.14 (0.93–1.38), 0.21	1.14 (0.88–1.48), 0.32
BASDAI >4, *n* (%)	-	0.57 (0.19–1.71), 0.31	0.39 (0.09–1.64), 0.20
BASFI, total score	-	1.27 (0.96–1.68), 0.10	1.38 (0.99–1.92), 0.058
PsAID, total score	-	1.21 (0.93-1.56), 0.16	1.22 (0.90–1.65), 0.20
DAPSA, total score	-	**1.12 (1.03–1.21), 0.010**	**1.15 (1.04–1.26), 0.004**
DAPSA categories	-		
Remission	-	-	-
Low	-	**10.73 (1.26–91.11), 0.030**	7.18 (0.78–66.46), 0.083
Moderate or high	-	**19.45 (1.86–203.55), 0.013**	11.01 (0.94–129.28), 0.056
BSA, total score	-	1.26 (0.95–7.74), 0.11	0.98 (0.61–1.57), 0.92
PASI, total score	-	1.36 (0.99–1.86), 0.057	1.31 (0.88–1.95), 0.18
NAPSI, total score	-	1.14 (0.96–1.35), 0.14	1.17 (0.97–1.41), 0.097
PGA, total score	-	1.81 (0.96–3.40), 0.066	1.67 (0.81–3.45), 0.17
Axial symptoms, *n* (%)	-	1.09 (0.52–2.25), 0.82	2.06 (0.74–5.76), 0.17
Peripheral symptoms, *n* (%)	-	0.45 (0.20–1.00), 0.051	0.62 (0.23–1.72), 0.36
Hip symptoms, *n* (%)	-	1.59 (0.69–3.65), 0.27	1.45 (0.48–4.37), 0.52
Enthesitis, *n* (%)	-	1.06 (0.51–2.19), 0.89	1.52 (0.59–3.92), 0.39
Uveitis, *n* (%)	-	1.15 (0.26–5.06), 0.85	0.48 (0.09–2.63), 0.40
Dactylitis, *n* (%)	-	1.30 (0.57–2.98), 0.53	0.58 (0.22–1.57), 0.28
Inflammatory bowel disease, *n* (%)	-	0.66 (0.12–3.62), 0.63	0.60 (0.08–4.75), 0.63
Sacroiliitis on MRI, *n* (%)	-	0.76 (0.24–2.37), 0.63	1.25 (0.34–4.57), 0.74
Syndesmophytes in axial X-ray, *n* (%)	-	0.99 (0.17–5.60), 0.99	0.88 (0.11–7.15), 0.90
Current NSAIDs, *n* (%)	-	1.13 (0.41–3.14), 0.81	1.53 (0.45–5.22), 0.50
Current prednisone, *n* (%)	-	1.52 (0.75–3.12), 0.25	1.52 (0.60–3.85), 0.37
DMARDs, *n* (%)	-	1.03 (0.44–2.41), 0.95	0.98 (0.31–3.14), 0.97
Methotrexate, *n* (%)	-	0.64 (0.30–1.36), 0.25	1.32 (0.49–3.53), 0.58
Anti TNF therapy, *n* (%)	-	0.87 (0.42–1.78), 0.69	1.46 (0.57–3.77), 0.43

Multinomial regression analyses are adjusted for age, sex and disease duration. Reference variable is 0 CV risk factors. Disease duration regression analysis with the number of CV risk factors is only adjusted for age and sex. In the analysis of the relation of DAPSA with the number of CV risk factor, remission category (-) is considered the reference category. CRP: C reactive protein; ESR: erythrocyte sedimentation rate; DMARD: disease-modifying antirheumatic drug BASDAI: Bath Ankylosing Spondylitis Disease Activity Index; BASFI: Bath Ankylosing Spondylitis Functional Index NAPSI: Nail Psoriasis Severity Index; BSA: Body Surface Area; PGA: Psoriasis Global Assessment; PsAID: PsA Impact of Disease Score DAPSA: Disease Activity in PSoriatic Arthritis; NSAIDs: Nonsteroidal anti-inflammatory drugs. DAPSA categories are remission, low, and moderate or high (3 categories). Remission is considered the reference category. *p* values lower than 0.05 are depicted in bold.
